# Supplementary material for: The geography of intergenerational social mobility in Britain
Source: Nat Commun. 2021 Oct 26;12:6050. doi: 10.1038/s41467-021-26185-z (PMC8548290; doi:10.1038/s41467-021-26185-z)
Supplement: Supplementary file 1 — Editor Summary [file 41467_2021_26185_MOESM1_ESM.docx]

Inter-generational preconditions and historical conferment of opportunity play a role in social mobility. This study considers the geography of relative deprivation to show how different family groups across Great Britain experience different inter-generational outcomes.
