# Supplementary material for: The geography of intergenerational social mobility in Britain
Source: Nat Commun. 2021 Oct 26;12:6050. doi: 10.1038/s41467-021-26185-z (PMC8548290; doi:10.1038/s41467-021-26185-z)
Supplement: Supplementary file 2 — Supplementary Information File [file 41467_2021_26185_MOESM2_ESM.pdf]

Supplementary Information for

The geography of inter-generational social mobility in Britain

Paul A. Longley*, Justin T. van Dijk, Tian Lan*

Department of Geography, University College London, Gower Street, London. WC1E 6BT.

*Corresponding authors: Paul A. Longley, p.longley@ucl.ac.uk; Tian Lan, tian.t.lan@ucl.ac.uk

Supplementary Tables

Supplementary Table 1. Number of individuals and coverage of the historical Censuses 1851-1911

Census Year	Number of Individuals	Geographic Coverage
1851	20,610,325	England, Wales, and Scotland
1861	22,837,378	England, Wales, and Scotland
1871	3,349,656	Scotland
1881	29,865,602	England, Wales, and Scotland
1891	33,522,822	England, Wales, and Scotland
1901	36,910,767	England, Wales, and Scotland
1911	36,353,455	England and Wales

Supplementary Figures

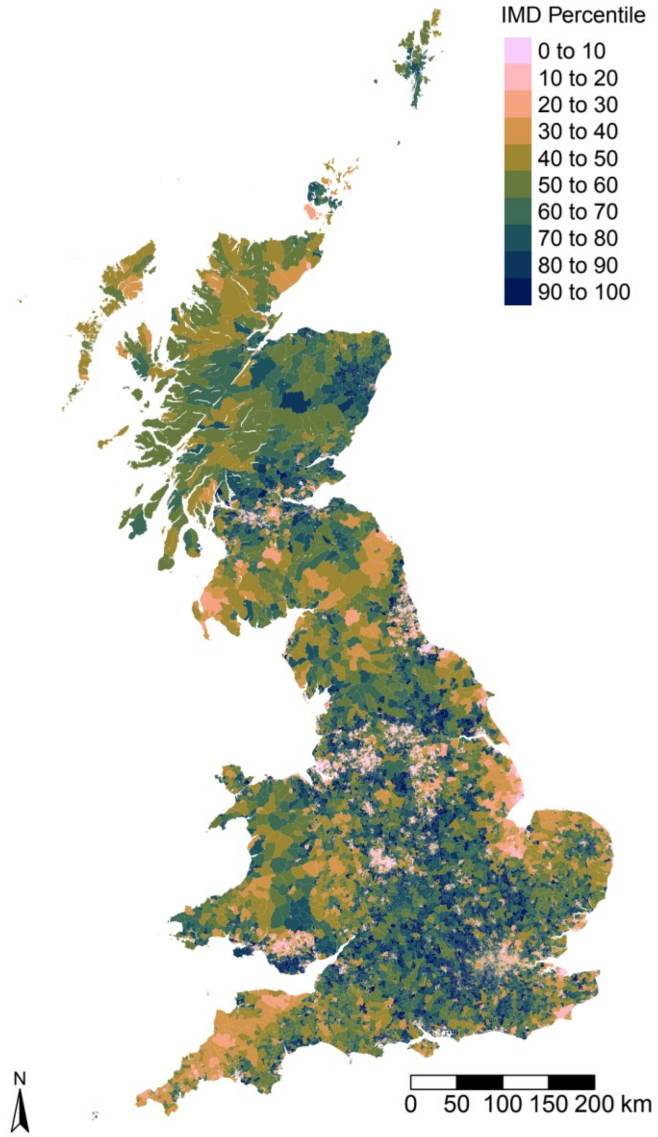

Supplementary Figure 1: Observed IMD scores. IMD percentile scores for England and Wales (2019) and Scotland (2020). Although the precise make-up of this composite measure differs between the countries that constitute the United Kingdom, the percentile scores can be considered broadly equivalent between neighbourhoods throughout Great Britain.

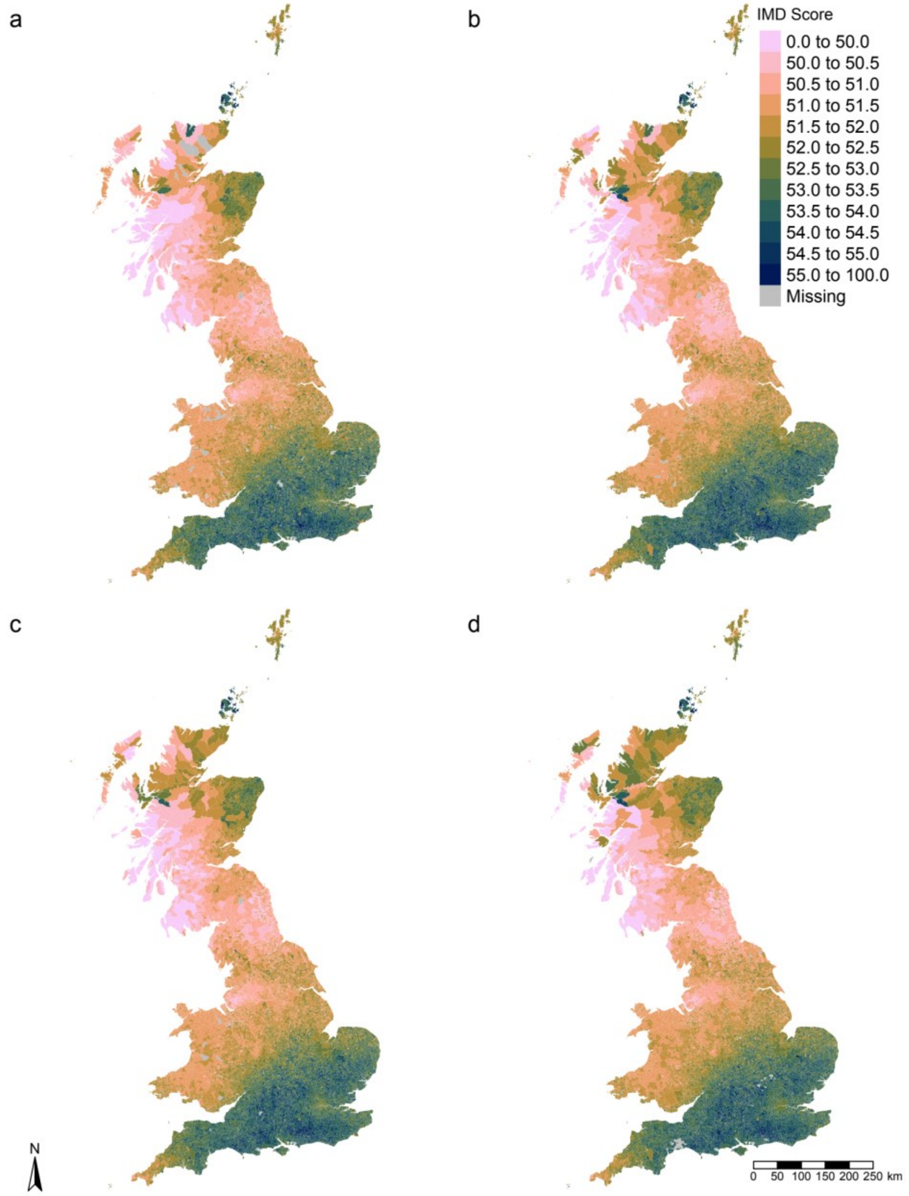

Supplementary Figure 2: Future IMD scores. The maps present the future IMD scores calculated using the **a** 1861, **b**1881, **c** 1891, and **d** 1901 historical Census data from the I-CeM collection. (Data for 1871 are not available from I-CeM.). The observed distributions closely resemble that shown in Figure 4.
