# Supplementary material for: The geography of intergenerational social mobility in Britain
Source: Nat Commun. 2021 Oct 26;12:6050. doi: 10.1038/s41467-021-26185-z (PMC8548290; doi:10.1038/s41467-021-26185-z)
Supplement: Supplementary file 4 — Reporting Summary [file 41467_2021_26185_MOESM4_ESM.pdf]

nature portfolio

Corresponding author(s): Paul Longley, Tian Lan

Last updated by author(s): Aug 17, 2021

Reporting Summary

Nature Portfolio wishes to improve the reproducibility of the work that we publish. This form provides structure for consistency and transparency in reporting. For further information on Nature Portfolio policies, see our Editorial Policies and the Editorial Policy Checklist.

Statistics

For all statistical analyses, confirm that the following items are present in the figure legend, table legend, main text, or Methods section.

n/a	Confirmed	
☐	☒	The exact sample size (*n*) for each experimental group/condition, given as a discrete number and unit of measurement
☒	☐	A statement on whether measurements were taken from distinct samples or whether the same sample was measured repeatedly
☐	☒	The statistical test(s) used AND whether they are one- or two-sided<br>*Only common tests should be described solely by name; describe more complex techniques in the Methods section.*
☒	☐	A description of all covariates tested
☒	☐	A description of any assumptions or corrections, such as tests of normality and adjustment for multiple comparisons
☐	☒	A full description of the statistical parameters including central tendency (e.g. means) or other basic estimates (e.g. regression coefficient) AND variation (e.g. standard deviation) or associated estimates of uncertainty (e.g. confidence intervals)
☒	☐	For null hypothesis testing, the test statistic (e.g. *F*, *t*, *r*) with confidence intervals, effect sizes, degrees of freedom and *P* value noted<br>*Give P values as exact values whenever suitable.*
☒	☐	For Bayesian analysis, information on the choice of priors and Markov chain Monte Carlo settings
☒	☐	For hierarchical and complex designs, identification of the appropriate level for tests and full reporting of outcomes
☐	☒	Estimates of effect sizes (e.g. Cohen's *d*, Pearson's *r*), indicating how they were calculated

Our web collection on statistics for biologists contains articles on many of the points above.

Software and code

Policy information about availability of computer code

Data collection	No software is used to collect the data.
Data analysis	All data analyses and computations are conducted with the standard R and Python packages. R version 4.1.0 was used for the analysis, using the data.table (1.14.0), raster (3.4.13), scico (1.2.0), sf (1.0.2), sparr (2.2.15), tidyverse (1.3.1), tmap (3.3.2) and tmaptools (3.3.1) packages. The birth parish to consistent parish string matching process was executed in Python (3.8.3) using the pandas (1.0.4), numpy (1.18.5), and rapidfuzz (0.9.1) libraries.

For manuscripts utilizing custom algorithms or software that are central to the research but not yet described in published literature, software must be made available to editors and reviewers. We strongly encourage code deposition in a community repository (e.g. GitHub). See the Nature Portfolio guidelines for submitting code & software for further information.

Data

Policy information about availability of data

All manuscripts must include a data availability statement. This statement should provide the following information, where applicable:

- Accession codes, unique identifiers, or web links for publicly available datasets
- A description of any restrictions on data availability
- For clinical datasets or third party data, please ensure that the statement adheres to our policy

The 2016 consumer register data (https://data.cdrc.ac.uk/dataset/linked-consumer-registers) and the I-CeM historical census data 1851-1911 (http://doi.org/10.5255/UKDA-SN-7856-2) used in this research are available upon successful application to the Consumer Data Research Centre (CDRC: cdrc.ac.uk) and the UK Data Service (UKDS: ukdataservice.ac.uk), respectively. The analysis conducted here was undertaken under special licensing arrangements for access to individual level data. As such, the data are only available upon successful application to these Economic and Social Research Council-funded data centres.

Field-specific reporting

Please select the one below that is the best fit for your research. If you are not sure, read the appropriate sections before making your selection.

☐ Life sciences ☒ Behavioural & social sciences ☐ Ecological, evolutionary & environmental sciences

For a reference copy of the document with all sections, see nature.com/documents/nr-reporting-summary-flat.pdf

Behavioural & social sciences study design

All studies must disclose on these points even when the disclosure is negative.

Study description	This research is a quantitative secondary data analysis study.
Research sample	The I-CeM historical census data 1851-1911 are full counts of the population. The 2016 consumer registers data are near-complete adult population of the UK. On average, the total number of records in the consumer registers for each year is only 1.8% different from the the Mid-year Population Estimates from 1997 to 2016, published by the Office for National Statistics UK.
Sampling strategy	No sampling strategy is adopted as the data are either full counts of the population or near-complete population.
Data collection	The data are secondary data and are collected by third parties.
Timing	The I-CeM historical census data 1851-1911 were collected every ten years. The consumer registers data are collected for every year between 1997 and 2016.
Data exclusions	Family names that have less than 100 bearers in 1851 and 200 bearers in 2016 are excluded from the analysis, which is described in the methodology section, as they might be misspelled.
Non-participation	The data are secondary data and are collected by third parties. There is no participant dropped out or declined.
Randomization	No experimental groups are in this study design hence randomization is not relevant in this study.

Reporting for specific materials, systems and methods

We require information from authors about some types of materials, experimental systems and methods used in many studies. Here, indicate whether each material, system or method listed is relevant to your study. If you are not sure if a list item applies to your research, read the appropriate section before selecting a response.

Materials & experimental systems

n/a	Involved in the study	
☒	☐	Antibodies
☒	☐	Eukaryotic cell lines
☒	☐	Palaeontology and archaeology
☒	☐	Animals and other organisms
☒	☐	Human research participants
☒	☐	Clinical data
☒	☐	Dual use research of concern

Methods

n/a	Involved in the study	
☒	☐	ChIP-seq
☒	☐	Flow cytometry
☒	☐	MRI-based neuroimaging
